# The Effect of Severity of Obstructive Sleep Apnea on Sleep Bruxism in Respiratory Polygraphy Study

**DOI:** 10.3390/brainsci12070828

**Published:** 2022-06-25

**Authors:** Klaudia Kazubowska-Machnowska, Anna Jodkowska, Monika Michalek-Zrabkowska, Mieszko Wieckiewicz, Rafal Poreba, Marzena Dominiak, Pawel Gac, Grzegorz Mazur, Justyna Kanclerska, Helena Martynowicz

**Affiliations:** 1 Department of Oral Surgery, Wroclaw Medical University, 26 Krakowska St., 50-425 Wroclaw, Poland; kkazubowska@gmail.com (K.K.-M.); marzenadominiak39@gmail.com (M.D.); 2 Department of Internal Medicine, Occupational Diseases, Hypertension and Clinical Oncology, Wroclaw Medical University, 213 Borowska St., 50-556 Wroclaw, Poland; anna.jodkowska@umw.edu.pl (A.J.); rafal.poreba@umw.edu.pl (R.P.); grzegorz.mazur@umw.edu.pl (G.M.); kanclerska.justyna@gmail.com (J.K.); helena.martynowicz@umw.edu.pl (H.M.); 3 Department of Experimental Dentistry, Wroclaw Medical University, 26 Krakowska St., 50-425 Wroclaw, Poland; mieszko.wieckiewicz@umw.edu.pl; 4 Department of Population Health, Division of Environmental Health and Occupational Medicine, Wroclaw Medical University, 7 Mikulicza-Radeckiego St., 50-345 Wroclaw, Poland; pawel.gac@umw.edu.pl

**Keywords:** polygraphy, obstructive sleep apnea, sleep bruxism, STOP-Bang questionnaire

## Abstract

Obstructive sleep apnea (OSA) and sleep bruxism (SB) may appear concomitantly. Data on the relationship between OSA and SB are limited. It was shown that in a population with an increased risk of OSA, OSA was dependently correlated with SB on the degree of OSA severity only in mild and moderate cases of OSA. We aimed to confirm this relationship and affecting factors in a group of dental office patients in a prospective, observational study. Adult patients (*n* = 119) were evaluated using respiratory polygraphy. The risk of OSA was assessed using a STOP-Bang questionnaire (SBQ). The episodes of bruxism and respiratory events were scored according to the standards of the American Academy of Sleep Medicine. The prevalence of OSA and SB was found to be 63.02% and 41.17%, respectively. The bruxism episode index (BEI) was increased in the group with a higher risk of OSA (SBQ ≥ 3) compared to the group with a lower risk of OSA (3.49 ± 3.63 vs. 2.27 ± 2.50, *p* = 0.03). The sensitivity and specificity of the SBQ were not sufficient to predict SB. A positive linear correlation between AHI and BEI in the group with AHI < 23/h was found. The study confirmed that OSA was associated with SB in the group of patients with OSA and/or SB risk. The relationship between OSA and SB depended on the degree of severity of OSA and occurred in mild and moderate cases of OSA.

## 1. Introduction

Obstructive sleep apnea (OSA) and sleep bruxism (SB) are both conditions of significant prevalence, occurring in up to 13% of the adult population for SB [1,2] and ranging from 9% to 38% for OSA [3]. In many cases, they may appear concomitantly. There were attempts devoted to assessing the relationship between OSA and sleep bruxism (SB), but data are limited and inconsistent [4,5,6,7,8,9,10,11]. However, Wieckiewicz et al. aimed to study the association of selected single-nucleotide polymorphisms (SNPs) occurring within the genes of the serotonin and dopamine pathways in individuals with SB and OSA [12]. The findings showed that HTR2A rs2770304 polymorphism might contribute to the association between SB and OSA.

OSA is the most common sleep disorder independently associated with cardiovascular morbidity and mortality [13,14,15,16,17]. It is characterized by airflow cessation due to the collapsing of the upper airways in the setting of a continued respiratory effort, leading to arterial oxygen desaturation, frequently terminated by arousal [3]. Sleep bruxism (SB) is a stereotyped behavior which is characterized by the rhythmic activity of jaw-muscles with grinding or clenching of the teeth during sleep and/or by the bracing or thrusting of the mandible [18]. According to the International Classification of Sleep Disorders (ICDS-3), the clinical criteria used for the diagnosis and classification of SB are as follows: regular or frequent tooth grinding sounds occurring during sleep (A) accompanied by, consistent with the above reports of sleep tooth grinding, at least one of the following clinical signs (B): (i) abnormal tooth wear and (ii) transient morning jaw-muscle pain or fatigue; and/or a temporal headache; and/or jaw locking upon awakening [19]. A definite, reliable sleep bruxism diagnosis is based on a combination of self-report, clinical inspection and polysomnography (preferably combined with audio/video recordings) [8].

Since pathophysiological data on SB are lacking [20], an investigation to clarify the nature of the relationship between SB and OSA may be necessary. Some studies on a few groups have failed to confirm the SB and OSA causative connection [7,8]; however, recently, the correlation has been demonstrated [9,10,11]. OSA has also been considered a new risk factor for SB, together with emotional stress, the consumption of tobacco, alcohol or coffee and anxiety disorders, which we recently confirmed [6,21,22,23]. In a previous study, we showed that OSA is correlated with SB in mild and moderate cases of OSA in a group of patients with an increased risk of OSA [21]. We aim to check if this connection could also be universal for other populations.

The aim of the present study is to assess the relationship between SB and OSA in a group of dental office patients with an OSA risk and SB suspicion, and to identify factors potentially affecting this relationship.

## 2. Materials and Methods

**Figure 1:**
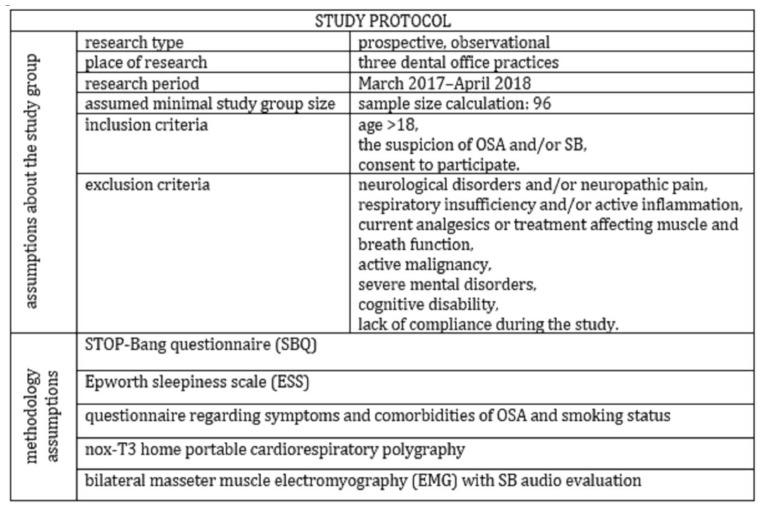
Flowchart of the study.

In this prospective, observational study, 119 adult patients with OSA and/or SB suspicion were enrolled between March 2017 and April 2018 in three dental clinics located in Wroclaw, Poland. The group size was estimated on the basis of a sample size calculator with the following assumptions: population size—2,800,000; fraction size—standard 0.5; maximum error—10%; confidence level—95%. The minimum required group size was 96 patients. The inclusion criteria obtained: age > 18, the suspicion of OSA and/or SB based both on self-reporting and clinical examination performed by experienced dentists and consent of the patient to participate in the study. We chose a dental practice, because we aimed to avoid bias because of comorbidity. The dental office population was younger and comorbidity (also concerning sleep disorders) was lower compared to sleep clinic patients. In the study, the following exclusion criteria were considered: confirmed neurological disorders and/or neuropathic pain, presence of respiratory insufficiency and/or active inflammation, current analgesics or treatment affecting muscle and breath function, confirmed active malignancy and severe mental disorders, as well as cognitive disability and lack of compliance during the study. The flowchart of the study is presented in Figure 1.

The risk of OSA was assessed using a STOP-Bang questionnaire (SBQ). Patients completed the STOP questions and answered four yes/no questions (BANG self-reported) about their body mass index (BMI), age, neck circumference and gender. The BMI was calculated as weight in kilograms divided by the square of height in meters. A STOP-Bang score of at least 3 is recommended to identify suspected OSA [24]. The Epworth sleepiness scale (ESS) was used to measure the subjects’ level of daytime sleepiness, with scores above 10 considered as excessive daytime sleepiness [25]. In addition to the ESS, the questionnaire collected data regarding symptoms and comorbidities of OSA and smoking status.

The Nox-T3 home portable cardiorespiratory polygraphy (Nox Medical, Iceland) was used in the study to collect data. Home portable polygraphy usefulness in SB and OSA diagnosis has been described in previous studies [26,27]. Heart rate, general activity and body position were collected as well as respiratory signals, such as snoring and nasal pressure, during the portable monitor recordings. Rib cage and abdominal movements were assessed with inductance plethysmography, and arterial oxygen saturation (SaO_2_) with finger pulse oximetry.

The standard criteria of the AASM Task Force were used to assess abnormal respiratory events. We defined apnea and hypopnea according to AASM guidelines [28].

SB was assessed using audio evaluation (an integrated microphone expansion device recorded sounds of bruxism) and bilateral masseter muscle electromyography (EMG). According to the AASM standards, bruxism episodes were grouped in phasic, tonic and mixed forms. To consider an EMG episode to be SB, at least doubling the amplitude of the background EMG activity was required. Gaps between EMG bursts belonging to the same SB episode should not have exceeded 3 s [28]. Additionally, at least two audible tooth-grinding episodes accompanying the EMG bursts were required to confirm bruxism diagnosis. Sleep BEI (bruxism episode index) standing for the number of bruxism episodes per hour of sleep was assumed, accordingly, to be irrelevant at <2, mild/moderate at 2–4 and >4 as severe [18].

In order to allow best credibility of diagnosis, collected data analysis and scoring were performed by the same specialist with extensive experience in sleep medicine, from the Sleep Laboratory at Wroclaw Medical University, Poland.

Statistical analysis was performed using the “Dell Statistica 13” software (Dell Inc., Aliso Viejo, CA, USA). Quantitative data are presented as mean values and standard deviations. Qualitative variables are expressed as a percentage. Significant statistical differences between arithmetic means were determined with the Mann–Whitney U test and between-group percentages with the chi-squared test. Evaluation of the test accuracy was performed based on the receiver–operator characteristic (ROC) curve analysis. Statistical significance was set at *p*-value < 0.05.

This study was approved by the Wroclaw Medical University Bioethics Committee (ID KB-195/2017). All subjects gave written informed consent in accordance with the Declaration of Helsinki. The Clinical Trial Registration identifier is: NCT03083405 (www.ClinicalTrials.gov, accessed on 1 April 2018).

## 3. Results

The mean age of all the participants was 50.90 ± 13.27 years. Women constituted 45.37% (*n* = 54) of all the participants. The mean BMI was found to be 26.35 ± 4.18 kg/m^2^. Smokers constituted 18.48% of all the participants. Diabetes and ischemic heart disease were diagnosed in 5.04% (*n* = 6) of the study population. The mean Epworth scale score was 7.36% ± 4.36 and the mean SBQ score was 3.41 ± 1.99. The increased risk of OSA (SBQ score ≥ 3) was found in 63.86% of the study population. Excessive daytime sleepiness (ESS > 10) was found in 26.05% *(n* = 31) of the study population.

The mean apnea–hypopnea index (AHI) and mean BEI were 12.16 ± 1.90 and 2.80 ± 1.90, respectively. The respiratory and bruxism parameters in the studied group are presented in Table 1.

The prevalence of OSA (AHI ≥ 5) was found to be 63.02% (*n* = 75) in the studied group. The SB (BEI ≥ 2) was diagnosed in 41.17% (*n* = 49) of the study population. The prevalence of mild, moderate and severe OSA and mild, moderate and severe SB is presented in Table 2.

The BEI was increased in the group with a higher risk of OSA (SBQ ≥ 3) compared with the group with a lower risk of OSA (3.49 ± 3.63 vs. 2.27 ± 2.50, *p* = 0.03). We also found a statistically significant correlation between BEI and the apnea index (OA/AI), mean saturation O_2_ (SpO_2_) and minimal SpO_2_ in the group with AHI < 5 (Table 3).

**Figure 2:**
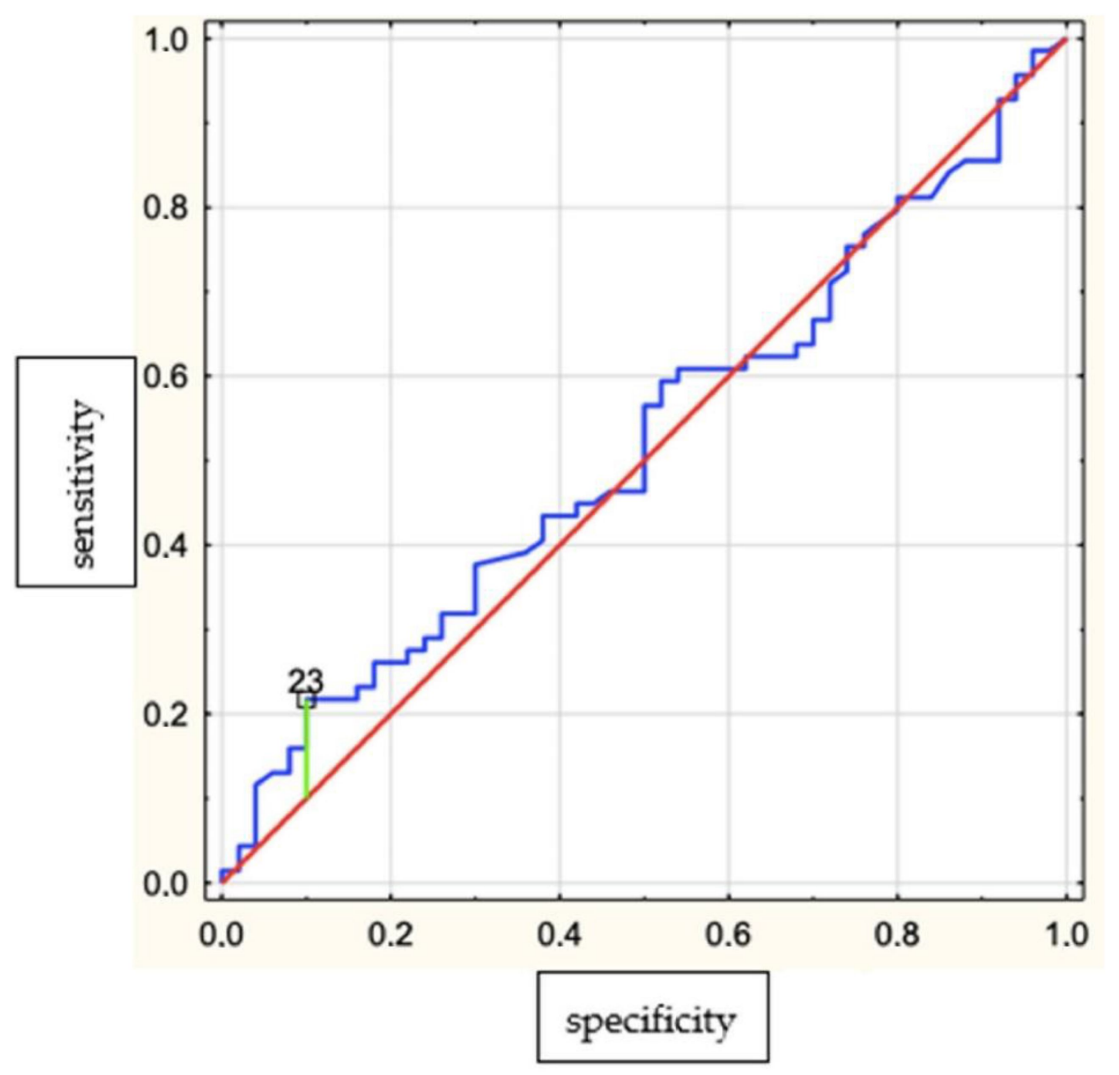
Receiver–operator characteristic (ROC) curve suggesting the optimal AHI cut-off point indicating its suitability for recognizing bruxism (BEI ≥ 2). Yauden’s index = 0.12.

We determined the ROC curve suggesting the optimal AHI cutoff point, indicating its suitability for recognizing bruxism (BEI ≥ 2). According to the course of the ROC curve, the cutoff point was set at AHI = 23 (Figure 2). A positive linear correlation between AHI and BEI in the group with AHI < 23 was found. The AHI cut-off point, 23 was selected based on the analysis of ROC curves as the criterion that best differentiated the predictive utility of AHI as a predictor of bruxism in this group of patients. There were 20 patients with AHI > 23.

**Figure 3:**
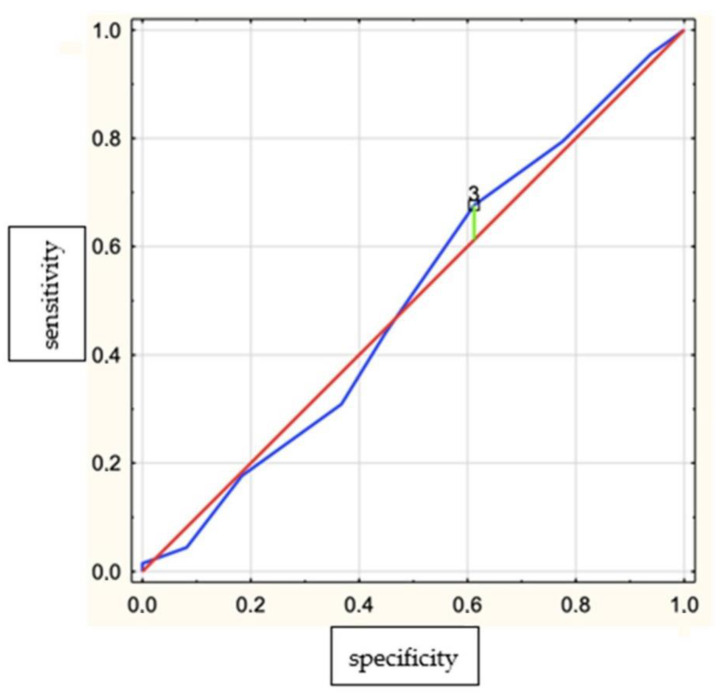
Receiver–operator characteristic (ROC) curves for the STOP-Bang for a cut-off point of ≥3. Yauden’s index = 0.06.

The correlation between bruxism parameters and respiratory parameters in the group with AHI < 23 and in the group with AHI > 23 is shown in Table 3. The sensitivity, specificity and accuracy of SBQ (SB > 3 for bruxism diagnosis BEI > 2) were 55.9%, 44.9% and 51.3%, respectively (Figure 3).

We did not find a statistically significant correlation between AHI and BEI in the whole studied group (*r* = −0.01, *p* > 0.05). No correlation between ESS and BEI was found (*r* = −0.02, *p* > 0.05). A positive correlation between AI (apnea index) and ESS was observed (*r* = 0.18, *p* < 0.05).

## 4. Discussion

The most interesting result of this study was the increased mean BEI in the group with an increased OSA risk measured with an SBQ compared to the group with a lower risk of OSA. It is worth noting that this questionnaire included questions about risk factors for bruxism, such as snoring or apneas [6], as well as new risk factors described recently by our research team, such as hypertension and BMI [27]. There is no simple and reliable tool to assess SB risk in clinical practice thus far. However, the sensitivity and specificity of the SBQ were not sufficient to predict SB. Some recently available data from the literature confirmed the genetic relationship between SB and OSA [12]. However, several studies suggested that SB appears more often in the OSA population than in healthy subjects [6,11], and some of them do not [7,8]. It is worth noting that many studies estimating the OSA–SB relationship were based on subjective methods of estimating bruxism, such as questionnaires, and no electromyography method was performed. Another common flaw in bruxism research is the small group of studied individuals.

We determined the ROC curve suggesting the optimal AHI cutoff point, indicating its suitability for recognizing bruxism (BEI ≥ 2). The cutoff point was established at AHI = 23. We showed a significant positive correlation between AHI and BEI in the group with AHI < 23. The correlation occurred in the group with milder disease, including mild OSA (AHI 5–15) and milder cases of moderate OSA (AHI < 23). It is noteworthy that no such correlation was found in patients with a more severe form of OSA (AHI > 23). This result indicated that the relationship between OSA and SB depended on the degree of severity of OSA. This was in agreement with our previous observation of patients in the sleep laboratory with a higher OSA risk [21] and other recent studies [11].

The data on the SB–OSA causative relationship are limited and inconsistent. One of the hypotheses is that SB activity could protect against OSA by protruding the mandible and restoring airway patency [26,27,29]. This could be a sort of secondary, not goal-oriented mechanism of prevention against OSA, plausibly not sufficient enough to prevent the airway from collapsing in more advanced stages of OSA. As we know from the human experimental model by Carra et al., sleep instability and arousal are suspected to be a permissive window for SB activity in predisposed individuals [9]. It was also previously hypothesized that rhythmic masticatory muscle activity is an autonomic reflex that may support the cerebral blood supply [10]. In some cases, the apnea could be interrupted by a contraction of the masseter muscles following arousal. In severe OSA, other mechanisms could be involved, leading to a reduction in SB episodes, such as excessive respiratory effort or increased respiratory rate following arousal, as well as changes in autonomic activity. It is worth noting that there was no further rise in the SB index reported in humans when sleep instability variables were experimentally increased [9]. This observation was parallel with the lack of an SB–OSA correlation in the severe OSA group in our study. Thus, the probable explanation of this phenomenon is the limited role of bruxism as a protective factor in severe OSA. Furthermore, a recent study by Wieczorek et al. concluded that sleep bruxism does not significantly affect sleep duration and efficiency; however, it changes sleep architecture and contributes to REM stage elongation. In this aforementioned study, SB did not affect respiratory parameters independently from SB severity [30]. However, both OSA and SB may share some parts of the pathophysiological pathway. Some hypotheses support the role of the central and autonomic nervous systems in the genesis of SB [31]. OSA is supposed to be connected to a higher sympathetic activity and there has been reports on the impaired brainstem inhibitory circuit control in sleep bruxism [32], as well as a positive association to serotonergic neurotransmission gene polymorphism [33]. Most SB episodes occur during cortical arousal associated with an increase in heart rate [34]. It has also been reported that preceding sleep deprivation and beta-blockers influence the BEI index in bruxers [10]. The homeostatic imbalance of the autonomic nervous system related to arousal during sleep constitutes the starting point for the cardiovascular implications of sleep bruxism. As has been previously discussed, increased sympathetic activity occurring during arousal leads to cardiovascular risk in SB patients. Cardiovascular consequences are also predominant in other sleep disturbances, for example, obstructive sleep apnea and insomnia.

Recently, the International Prospective Urban Rural Epidemiology (PURE) study showed that over half of the Polish general population had an increased risk of OSA (66.5% of men and 60.1% of women) [35]. In our study, the increased risk of OSA (SBQ score ≥ 3) was found in 63.86%. Thus, the studied population had an incidence of OSA closer to the populational level, which makes the conclusion more in agreement with the general population. Support for our findings was presented by the study of Tsujisakaa et al., where 22 SB patients, primarily asymptomatic in the context of OSA, were diagnosed with polysomnography. In total, 27% of patients was diagnosed with mild-level OSA and reported a positive correlation of RMMA and NSMA (non-specific masticatory activity), occurring closely to respiratory events with the apnea–hypopnea index (AHI) [11].

Data on bruxism and sleepiness are very limited. Increased sleepiness, as measured by ESS, was described in bruxers; however, the association of sleepiness with OSA was not demonstrated in earlier studies [36]. Neu et al. showed that 22 patients with SB presented higher levels of daytime fatigue and sleepiness than 12 control subjects; thus, the studied group was not enough [37]. In our study, there were no statistical differences found between bruxers (BEI ≥ 2) and non-bruxers (BEI < 2) in a level of daytime sleepiness. It needs to be emphasized that we used cardiorespiratory polygraphy and masseter muscle EMG with audio analyses, which are objective methods used for respiratory disturbance and bruxism diagnosis. There was no association between sleepiness, measured as ESS, and BEI; however, an association between ESS and AI was found. Excessive daytime sleepiness is one of the classic symptoms of OSA [38]. OSA is considered a risk factor for SB; it has been observed that increased sleepiness in SB may actually be a result of the co-occurrence of undiagnosed OSA. The hypothesis is in agreement with a recent study [11].

This study had a few limitations. We did not use polysomnography, which is considered the gold standard in the diagnosis of SB. However, the device used in this study was validated for the diagnosis of OSA, and its diagnostic accuracy in SB diagnosis can be increased by the addition of audio and masseter EMG signals. Another limitation was the lack of randomization in the selection of the patients who were asked to participate in the study.

In summary, we confirmed the OSA–SB correlation both in the sleep laboratory [21] and dental office populations of patients in the present study. Both studies showed the effect of OSA severity on OSA–SB incidence, which could explain the discrepancy in previous studies’ results. Therefore, clinicians should take into account the increased risk of SB in patients with mild and moderate OSA, and these patients should receive the care of both a sleep specialist and a dentist.

The present findings confirmed the OSA–SB positive correlation in populations with different risks of OSA [11,21], and gave rise to new questions about the nature of the causative connection between SB and OSA. One of the plausible hypotheses is that SB could be extinguished with the severity of OSA. The explanation of whether a direct mechanism may be associated with the neurological mechanism of the induction and blockade or rather connected with the anatomical location and quantitative area of the airway’s obstruction needs further investigation.

## 5. Conclusions

According to the results of this study, sleep bruxism intensity was increased in patients with an increased risk of OSA and OSA was associated with SB. The relationship between OSA and SB occurred in mild and moderate OSA. Due to an increased intensity of SB, special oral care is needed in patients with mild and moderate OSA. Further work is certainly required to disentangle the role of primary and secondary sleep bruxism.

## Figures and Tables

**Table 1:** The respiratory and bruxism indexes in studied group.

Parameter	Studied Group (*n* = 119)	SBQ ≥ 3 (*n* = 52)	SBQ < 3 (*n* = 52)	*p*
BEI (*n*/hour)	2.80 ± 3.08	3.39 ± 3.63	2.27 ± 2.50	0.03 *
Phasic (*n*/hour)	1.00 ± 1.54	1.30 ± 1.76	0.77 ± 1.33	0.07
Tonic (*n*/hour)	0.92 ± 1.08	1.12 ± 1.38	0.76 ± 0.77	0.08
Mixed (*n*/hour)	0.89 ± 1.18	1.07 ± 1.40	0.74 ± 0.98	0.14 *
AHI (*n*/hour)	12.16 ± 13.90	21.19 ± 16.05	5.19 ± 5.74	0.00 **
ODI (*n*/hour)	12.53 ± 13.39	21.40 ± 15.26	5.71 ± 5.61	0.00 **
Snore (%)	10.21 ± 15.13	16.43 ± 17.80	5.48 ± 10.64	0.00 **
OA *(n*/hour)	4.16 ± 8.10	8.15 ± 10.77	1.07 ± 2.36	0.00 **
MA (*n*/hour)	0.24 ± 0.82	0.52 ± 1.19	0.03 ± 0.08	0.00 **
CA (*n*/hour)	0.73 ± 1.23	0.99 ± 1.58	0.50 ± 0.83	0.03 *
Hypopnea (*n*/hour)	7.04 ± 6.77	11.52 ± 7.18	3.61 ± 3.80	0.00 **
Cheyne–Stokes (%)	0.21 ± 1.55	0.48 ± 2.34	0.00 ± 0.00	0.10
Mean SpO_2_ (%)	93.70 ± 1.84	92.71 ± 1.73	94.48 ± 1.53	0.00 **
Min SO_2_ (%)	85.61 ± 5.36	82.87 ± 5.05	87.68 ± 4.65	0.00 **
SpO_2_ < 90% (%)	5.47 ± 13.24	9.86 ± 17.44	2.11 ± 7.27	0.00 *
Mean oxygen desaturation (%)	4.09 ± 1.29	4.65 ± 1.49	3.64 ± 0.91	0.00 *
Mean heart rate (beats/minute)	62.26 ± 7.48	63.01 ± 8.01	61.93 ± 6.88	0.43
Max heart rate (beats/minute)	97.48 ± 16.30	93.58 ± 10.56	100.72 ± 19.43	0.02 *
Min heart rate (beats/minute)	48.52 ± 7.17	48.48 ± 8.00	48.78 ± 6.47	0.77

BEI—bruxism episode index; AHI—apnea–hypopnea index; OA—obstructive apnea; MA—mixed apnea; CA—central apnea; ODI—oxygen desaturation index; SpO_2_ < 90% (%)—time with oxygen saturation below 90% (% total sleep time); * *p* < 0.05; ** *p* < 0.01.

**Table 2:** The prevalence of obstructive sleep apnea and sleep bruxism in studied group.

Parameter		%	*n*
AHI (*n*/hour)	<5	36.97	44
	≥5 < 15	37.81	45
	≥15 < 30	11.76	14
	≥30	12.60	15
BEI (*n*/hour)	<2	58.82	70
	≥2 < 4	18.48	22
	≥4	27.73	33

BEI—bruxism episode index; AHI—apnea–hypopnea index.

**Table 3:** The correlations between respiratory indices and BEI in subgroups.

Group	AHI < 5	AHI <23	AHI > 23
Parameters	BEI (*n*/hour)		
AHI *(n*/hour)	0.10	0.22 *	0.05
ODI (*n*/hour)	−0.07	0.19	0.06
Snore (%)	−0.07	0.01	0.48 *
OA (*n*/hour)	0.42 *	0.18	0.03
MA (*n*/hour)	0.00	0.20 *	−0.05
CA (*n*/hour)	0.10	0.14	0.06
Hypopnea (*n*/hour)	−0.10	0.17	0.04
Mean SpO_2_ (%)	0.33 *	−0.07	0.08
Min SpO_2_ (%)	0.35 *	−0.12	0.10
SpO_2_ < 90% (%)	0.15	0.30 *	−0.07
Mean oxygen desaturation (%)	−0.10	0.09	−0.02
Mean heart rate (beats/minute)	−0.06	0.05	−0.06
Max heart rate (beats/minute)	−0.08	0.02	−0.00
Min heart rate (beats/minute)	−0.23	−0.24	0.02

BEI—bruxism episode index; AHI—apnea–hypopnea index; OA—obstructive apnea; MA—mixed apnea; CA—central apnea; ODI—oxygen desaturation index; SpO_2_ < 90% (%)—time with oxygen saturation below 90% (% of total sleep time); * *p* < 0.05.

## Data Availability

Not applicable.

## References

[1] Lobbezoo F., Ahlberg J., Raphael K.G., Wetselaar P., Glaros A.G., Kato T., Santiago V., Winocur E., De Laat A., De Leeuw R. (2018). International consensus on the assessment of bruxism: Report of a work in progress. J. Oral Rehabil..

[2] Kato T., Velly A.M., Nakane T., Masuda Y., Maki S. (2012). Age is associated with self-reported sleep bruxism, independently of tooth loss. Sleep Breath.

[3] Hollowell D.E., Bhandary P.R., Funsten A.W., Suratt P.M. (1991). Respiratory related recruitment of the masseter: Response to hypercapnia and loading. J. Appl. Physiol..

[4] Lévy P., Kohler M., McNicholas W.T., Barbé F., McEvoy R.D., Somers V.K., Lavie L., Pépin J.-L. (2015). Obstructive sleep apnea syndrome. Nat. Rev. Dis. Primers..

[5] Jokubauskas L., Baltrušaitytė A. (2017). Relationship between obstructive sleep apnoea syndrome and sleep bruxism: A systematic review. J. Oral Rehabil..

[6] Hosoya H., Kitaura H., Hashimoto T., Ito M., Kinbara M., Deguchi T., Irokawa T., Ohisa N., Ogawa H., Takano-Yamamoto T. (2014). Relationship between sleep bruxism and sleep respiratory events in patients with obstructive sleep apnea syndrome. Sleep Breath.

[7] Saito M., Yamaguchi T., Mikami S., Watanabe K., Gotouda A., Okada K., Hishikawa R., Shibuya E., Shibuya Y., Lavigne G. (2016). Weak association between sleep bruxism and obstructive sleep apnea. A sleep laboratory study. Sleep Breath.

[8] Sjöholm T.T., Lowe A.A., Miyamoto K., Fleetham J.A., Ryan C.F. (2000). Sleep bruxism in patients with sleep-disordered breathing. Arch. Oral Biol..

[9] Carra M.C., Rompre P.H., Kato T., Parrino L., Terzano M.G., Lavigne G.J., Macaluso G.M. (2011). Sleep bruxism and sleep arousal: An experimental challenge to assess the role of cyclic alternating pattern. J. Oral Rehabil..

[10] Sjöholm T., Polo O., Mäntyvaara J., Tanner J., Piha J., Lehtinen I. (1996). Does sleep bruxism serve a physiological purpose?. Electroencephalogr. Clin. Neurophysiol..

[11] Tsujisakaa A., Harakia S., Nonouebd S., Mikamib A., Adachib H., Mizumoria T., Yatania H., Yoshidae A., Katobe T. (2018). The occurrence of respiratory events in young subjects with a frequent rhythmic masticatory muscle activity: A pilot study. J. Prosthodont. Res..

[12] Wieckiewicz M., Bogunia-Kubik K., Mazur G., Danel D., Smardz J., Wojakowska A., Poreba R., Dratwa M., Chaszczewska-Markowska M., Winocur E. (2020). Genetic basis of sleep bruxism and sleep apnea—Response to a medical puzzle. Sci. Rep..

[13] Hou H., Zhao Y., Yu W., Dong H., Xue X., Ding J., Xing W., Wang W. (2018). Association of obstructive sleep apnea with hypertension: A systematic review and meta-analysis. J. Glob. Health..

[14] Munoz R., Duran-Cantolla J., Martinez-Vila E., Gallego J., Rubio R., Aizpuru F., De La Torre G. (2006). Severe sleep apnea and risk of ischemic stroke in the elderly. Stroke.

[15] Vasheghani-Farahani A., Kazemnejad F., Sadeghniiat-Haghighi K., Saadat S., Tavakolipoor P., Yazdani T., Alidoosti M., Ghasem-Amooeian V., Ashraf H. (2018). Obstructive sleep apnea and severity of coronary artery disease. Caspian J. Intern. Med..

[16] Mehra R., Benjamin E.J., Shahar E., Gottlieb D.J., Nawabit R., Kirchner H., Sahadevan J., Redline S. (2006). Sleep Heart Health Study Association of nocturnal arrhythmias with sleep-disordered breathing: The Sleep Heart Health Study. Am. J. Respir Crit Care Med..

[17] Young T., Finn L., Peppard P.E. (2008). Sleep disordered breathing and mortality: Eighteen-year follow-up of the Wisconsin sleep cohort. Sleep.

[18] American Academy of Sleep Medicine (2014). International Classification of Sleep Disorders.

[19] Sateia M.J. (2014). International classification of sleep disorders-third edition: Highlights and modifications. Chest.

[20] Bader G., Lavigne G. (2000). Sleep bruxism; an overview of an oromandibular sleep movement disorder. Sleep Med. Rev..

[21] Martynowicz H., Gac P., Brzecka A., Poreba R., Wojakowska A., Mazur G., Smardz J., Wieckiewicz M. (2019). The Relationship between Sleep Bruxism and Obstructive Sleep Apnea Based on Polysomnographic Findings. J. Clin. Med..

[22] Kuhn M., Türp J.C. (2018). Risk factors for bruxism. Swiss. Dent. J..

[23] Ohayon M.M., Li K.K., Guilleminault C. (2001). Risk factors for sleep bruxism in the general population. Chest.

[24] Nagappa M., Wong J., Singh M., Wong D.T., Chung F. (2017). An update on the various practical applications of the STOP-Bang questionnaire in anesthesia, surgery, and perioperative medicine. Curr. Opin. Anaesthesiol..

[25] Johns M.W. (1991). A new method for measuring daytime sleepiness: The Epworth sleepiness scale. Sleep.

[26] Winck M., Drummond M., Viana P., Pinho J.C., Winck J.C. (2017). Sleep bruxism associated with obstructive sleep apnoea syndrome—A pilot study using a new portable device. Rev. Port. Pneumol..

[27] Martynowicz H., Dymczyk P., Dominiak M., Kazubowska K., Skomro R., Poreba R., Gac P., Wojakowska A., Mazur G., Wieckiewicz M. (2018). Evaluation of Intensity of Sleep Bruxism in Arterial Hypertension. J. Clin. Med..

[28] Berry R.B., Brooks R., Gamaldo C., Harding S.M., Lloyd R.M., Quan S.F., Troester M.T., Vaughn B.V., American Academy of Sleep Medicine (2017). The AASM Manual for the Scoring of Sleep and Associated Events: Rules, Terminology and Technical Specifications.

[29] Manfredini D., Guarda-Nardini L., Marchese-Ragona R., Lobbezoo F. (2015). Theories on possible temporal relationships between sleep bruxism and obstructive sleep apnea events. An expert opinion. Sleep Breath.

[30] Wieczorek T., Wieckiewicz M., Smardz J., Wojakowska A., Michalek-Zrabkowska M., Mazur G., Martynowicz H. (2020). Sleep structure in sleep bruxism: A polysomnographic study including bruxism activity phenotypes across sleep stages. J. Sleep Res..

[31] Klasser G.D., Rei N., Lavigne G.J. (2015). Sleep bruxism etiology: The evolution of a changing paradigm. J. Can. Dent. Assoc..

[32] Inana R., Benbir G., Karadeniz D., Yavlal F., Kiziltanb M.E. (2017). Sleep bruxism is related to decreased inhibitory control of trigeminal motoneurons, but not with reticulobulbar system. Neurol. Sci..

[33] Abe Y., Suganuma T., Ishii M., Yamamoto G., Gunji T., Clark G.T., Tachikawa T., Kiuchi Y., Igarashi Y., Baba K. (2012). Association of genetic, psychological and behavioral factors with sleep bruxism in a Japanese population. J. Sleep Res..

[34] Lavigne G., Manzini C., Huynh N.T., Kryger M.H., Roth T., Dement W.C. (2011). Sleep bruxism. Principles and Practice of Sleep Medicine.

[35] Postrzech-Adamczyk K., Nahorecki A., Zatońska K., Lawson J., Wołyniec M., Skomro R., Szuba A. (2019). Prevalence and Risk of Obstructive Sleep Apnea and Arterial Hypertension in the Adult Population in Poland: An Observational Subset of the International Prospective Urban Rural Epidemiology (PURE) Study. Adv. Exp. Med. Biol..

[36] Câmara-Souza M.B., de Figueredo O.M.C., Rodrigues Garcia R.C.M. (2018). Association of sleep bruxism with oral health-related quality of life and sleep quality. Clin. Oral Investig..

[37] Neu D., Baniasadi N., Newell J., Styczen D., Glineur R., Mairesse O. (2018). Effect of sleep bruxism duration on perceived sleep quality in middle-aged subjects. Eur. J. Oral Sci..

[38] Stansbury R.C., Strollo P.J. (2015). Clinical manifestations of sleep apnea. J. Thorac. Dis..

